# Work of Breathing into Snow in the Presence versus Absence of an Artificial Air Pocket Affects Hypoxia and Hypercapnia of a Victim Covered with Avalanche Snow: A Randomized Double Blind Crossover Study

**DOI:** 10.1371/journal.pone.0144332

**Published:** 2015-12-14

**Authors:** Karel Roubík, Ladislav Sieger, Karel Sykora

**Affiliations:** 1 Department of Biomedical Technology, Faculty of Biomedical Engineering, Czech Technical University in Prague, Prague, Czech Republic; 2 Department of Physics, Faculty of Electrical Engineering, Czech Technical University in Prague, Prague, Czech Republic; 3 Department of Physiology and Biochemistry, Faculty of Physical Education and Sport, Charles University, Prague, Czech Republic; Erasmus Medical Centre, NETHERLANDS

## Abstract

Presence of an air pocket and its size play an important role in survival of victims buried in the avalanche snow. Even small air pockets facilitate breathing. We hypothesize that the size of the air pocket significantly affects the airflow resistance and work of breathing. The aims of the study are (1) to investigate the effect of the presence of an air pocket on gas exchange and work of breathing in subjects breathing into the simulated avalanche snow and (2) to test whether it is possible to breathe with no air pocket. The prospective interventional double-blinded study involved 12 male volunteers, from which 10 completed the whole protocol. Each volunteer underwent two phases of the experiment in a random order: phase “AP”—breathing into the snow with a one-liter air pocket, and phase “NP”—breathing into the snow with no air pocket. Physiological parameters, fractions of oxygen and carbon dioxide in the airways and work of breathing expressed as pressure-time product were recorded continuously. The main finding of the study is that it is possible to breath in the avalanche snow even with no air pocket (0 L volume), but breathing under this condition is associated with significantly increased work of breathing. The significant differences were initially observed for end-tidal values of the respiratory gases (EtO_2_ and EtCO_2_) and peripheral oxygen saturation (SpO_2_) between AP and NP phases, whereas significant differences in inspiratory fractions occurred much later (for F_I_O_2_) or never (for F_I_CO_2_). The limiting factor in no air pocket conditions is excessive increase in work of breathing that induces increase in metabolism accompanied by higher oxygen consumption and carbon dioxide production. The presence of even a small air pocket reduces significantly the work of breathing.

## Introduction

Avalanche burials represent one of the most dangerous risks associated with winter activities in the mountains. According to Falk et al. [1], after 15 minutes under the avalanche snow the survival probability is 92%, and the survival rate drops precipitously to 30% in 35 minutes. Haegeli et al. [2] describes that the first 10 minutes might be more appropriate general guideline for Canada and other areas with a maritime snow climate. Acute asphyxiation is the dominant cause of avalanche-related deaths [3–5]. Asphyxiation occurs as a consequence of blocked airways, or, due to a severe hypoxia and hypercapnia resulting from rebreathing previously exhaled gas. However, snow is a porous material and if the exhaled gas is diverted from the space of gas suction using a specific device, a person buried in snow is able to sustain breathing longer than 60 minutes [6–8] or even 90 minutes [9].

When a victim repetitively inspires previously expired gas, the inhaled gas gradually contains less oxygen and higher concentration of carbon dioxide. The speed of this process is thought to depend on the volume of air in the pocket. Paal et al. [10] conducted an experiment in piglets covered by snow and breathing into the air pockets of 1 L or 2 L volumes within the snow mass, or breathing ambient air. The aim of their study was, using a porcine model, to investigate the combined effects of hypothermia, hypoxia and hypercapnia in a realistic avalanche burial scenario. Since the study was terminated prematurely, it was not possible to evaluate differences in effects of 1 L and 2 L air pockets. Brugger et al. [11] conducted another experiment in 12 volunteers breathing into snow pockets of volume 1 L or 2 L. The decrease in peripheral oxygen saturation (SpO_2_) within 4 minutes was significantly greater in tests with the smaller air pocket. Furthermore, they observed a highly significant inverse correlation between the decrease in SpO_2_ and time to interruption or completion.

Presently, an accompanying air pocket is defined as any detectable air space around the face with the proviso of a patent airway [12, 13]. To our knowledge, a study investigating the effects of breathing with no air pockets has not yet been published.

Therefore, the purpose of the current study was to compare the snow pockets of two volumes of air: 0 L (“no air pocket”) and 1 L (“air pocket”) in front of patent nose and oral cavity. Considering the small amount of oxygen present in the 1 L air pocket (210 mL) and the physiological oxygen demands of the organism, we hypothesize that 1 L air pocket should not have a significant effect on the oxygen supply and carbon dioxide removal in comparison with the no air pocket. During our pilot experiments we observed that subjects breathing under hypercapnia (end-tidal partial pressure of CO_2_ > 55 mm Hg) developed tidal volumes over 3 L, which are much larger than the volumes of air pockets in the previously published studies. We believe that the main effect of the air pockets smaller than the tidal volumes is less dependent on providing spare air for breathing. Instead, we hypothesize that the size of the air pocket significantly affects the airflow resistance and work of breathing. A victim buried in the snow must overcome these obstacles by increased work of respiratory muscles. Substantial increases in work of breathing may have a significant effect on the elevation of the total metabolic rate of the organism [14] leading to an increased consumption of oxygen and increased production of carbon dioxide. These effects further deteriorate the composition of arterial blood gases and the overall physiological state of the victim covered with avalanche snow.

The aim of the present study was to investigate the effect of the air pocket volume in front of patent nose and oral cavity on gas exchange and work of breathing in subjects breathing into a simulated avalanche snow, and to test whether it is possible to breathe with no air pocket.

## Methods

The study protocol was approved by the Institutional Review Board of the Faculty of Physical Education and Sport, Charles University in Prague (No. 077/2012, issued on February 17, 2012, S1 File), and written informed consent was obtained from the volunteers before enrollment in the study. The study registration number in the ClinicalTrials.gov register is NCT02521272. The study was not registered prior to the enrolment of the participants as the registration is not required by the European Union authorities for this study type. The authors confirm that all ongoing and related trials for this intervention are registered.

The prospective interventional randomized double-blinded crossover study was conducted in Spindleruv Mlyn, Krkonose Mts., Czech Republic, at the altitude of 762 meters above sea level on March 4–9, 2012.

### Subjects

**Fig 1:**
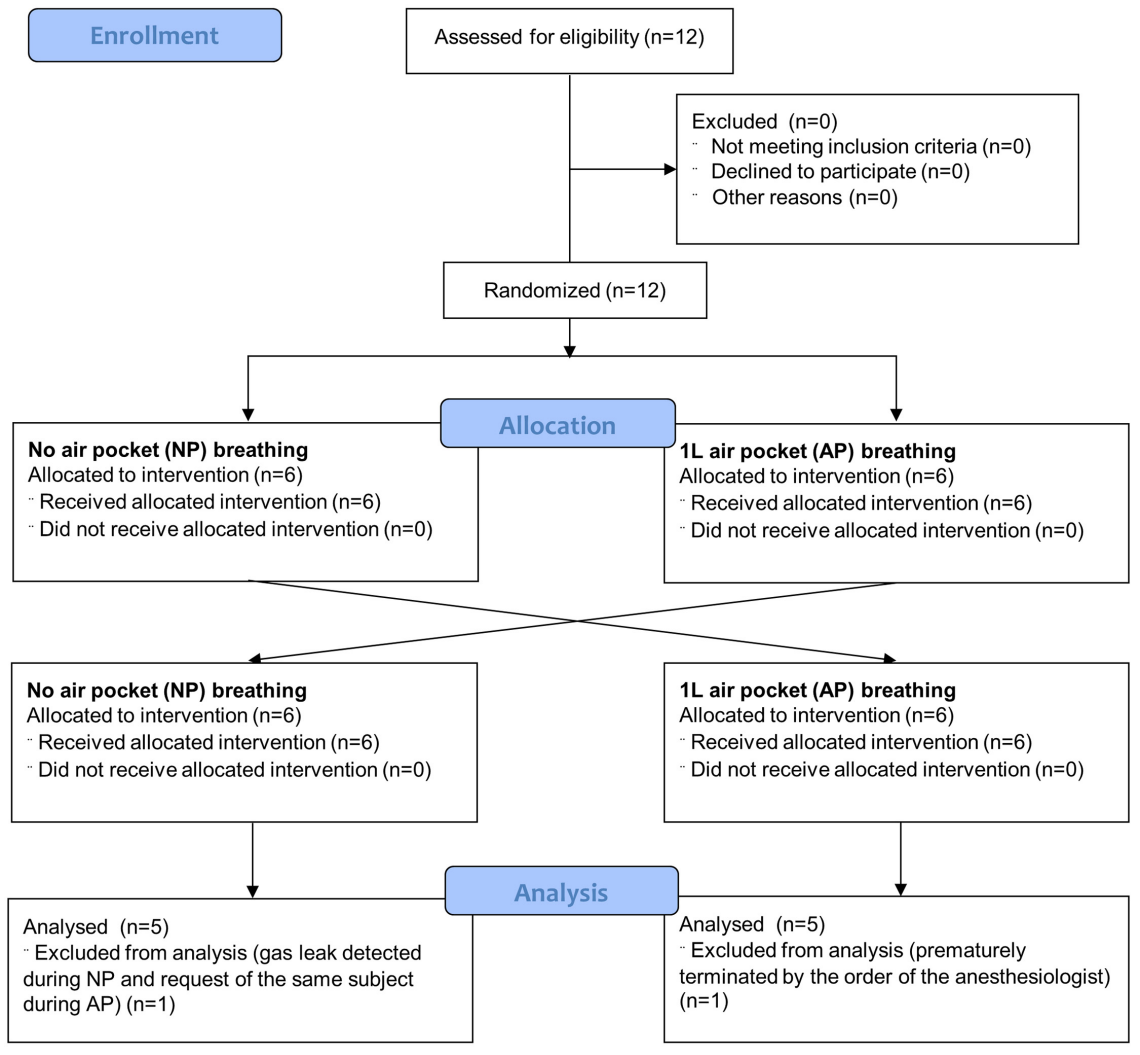
Flow diagram of the study with enrollment, allocation and analysis of the participants.

Twelve male volunteers participated in the study, from which 10 completed the protocol and were included in the evaluation (Fig 1). The number of subjects included in the study was selected as a maximum number of subjects included in the published studies with a similar scope and design [6, 7, 8, 9, 11]. The participant recruitment and their participation in the study were conducted on March 4–9, 2012. The characteristics of the study group are presented in Table 1. Eligible participants were volunteers from the Czech Army forces, studying at the Military Department of the Faculty of Physical Education and Sport, Charles University in Prague. All subjects were healthy and fit, classified according to the American Society of Anesthesiologists as ASA I [15], all without a smoking history. The volunteers were highly motivated to participate in the experiment. The entrance examination, completed before the start of the study, included these tests: electrocardiography, blood pressure, spirometry (MSP1, Mesit, Uherske Hradiste, CZ), and assessment of the health conditions and family anamnesis by a physician with a specialty in anesthesia and critical care. The exclusion criteria were Tiffeneau Index less than 0.70 and any cardiovascular or respiratory condition.

**Table 1:** The basic characteristics of the group of volunteers involved in the breathing experiment.

Parameter	All volunteers (N = 12)	Volunteers completed the protocol (N = 10)
Age (years)	24.8 ± 3.4 (20–30)	24.6 ± 3.5 (20 – 30)
Weight (kg)	77.7 ± 7.1 (64 – 90)	76.1 ± 6.4 (64 – 85)
Height (cm)	180.0 ± 5.5 (173 – 192)	179.3 ± 5.8 (173–192)
BMI (kg m^–2^)	24.0 ± 1.7 (20–27)	23.7 ± 1.6 (20–25)
FEV1 (L)	4.5 ± 0.4 (4.0–5.1)	4.4 ± 0.4 (4.0–4.9)
FVC (L)	5.2 ± 0.5 (4.5–6.1)	5.2 ± 0.5 (4.5–6.1)

The values are presented as mean ± standard deviation and range (minimum–maximum). Abbreviations: BMI—Body Mass Index; FEV1—Forced Expiratory Volume in 1 second; FVC—Forced Vital Capacity.

### Study design

Each volunteer underwent two phases of the experiment: phase “AP”—breathing into the snow with a one-liter air pocket, and phase “NP”—breathing into the snow with no air pocket. Volunteers were assigned to the AP and NP phases in random order based on the balanced blocked randomization (block size = 12, two arms, drawing preprinted randomization papers from a raffle drum) conducted by an independent assistant. Neither the volunteers nor the investigators knew whether the prepared experiment involved breathing with AP or NP. Only the principal investigator and two assistants preparing the AP or NP arrangement in the snow knew this information. All the subjects stayed at the altitude of the experiment at least for 48 hours before the experiment and they rest at the same place until the whole experiment was completed. At least a 20-hour recovery interval was inserted between the two phases in each subject.

**Fig 2:**
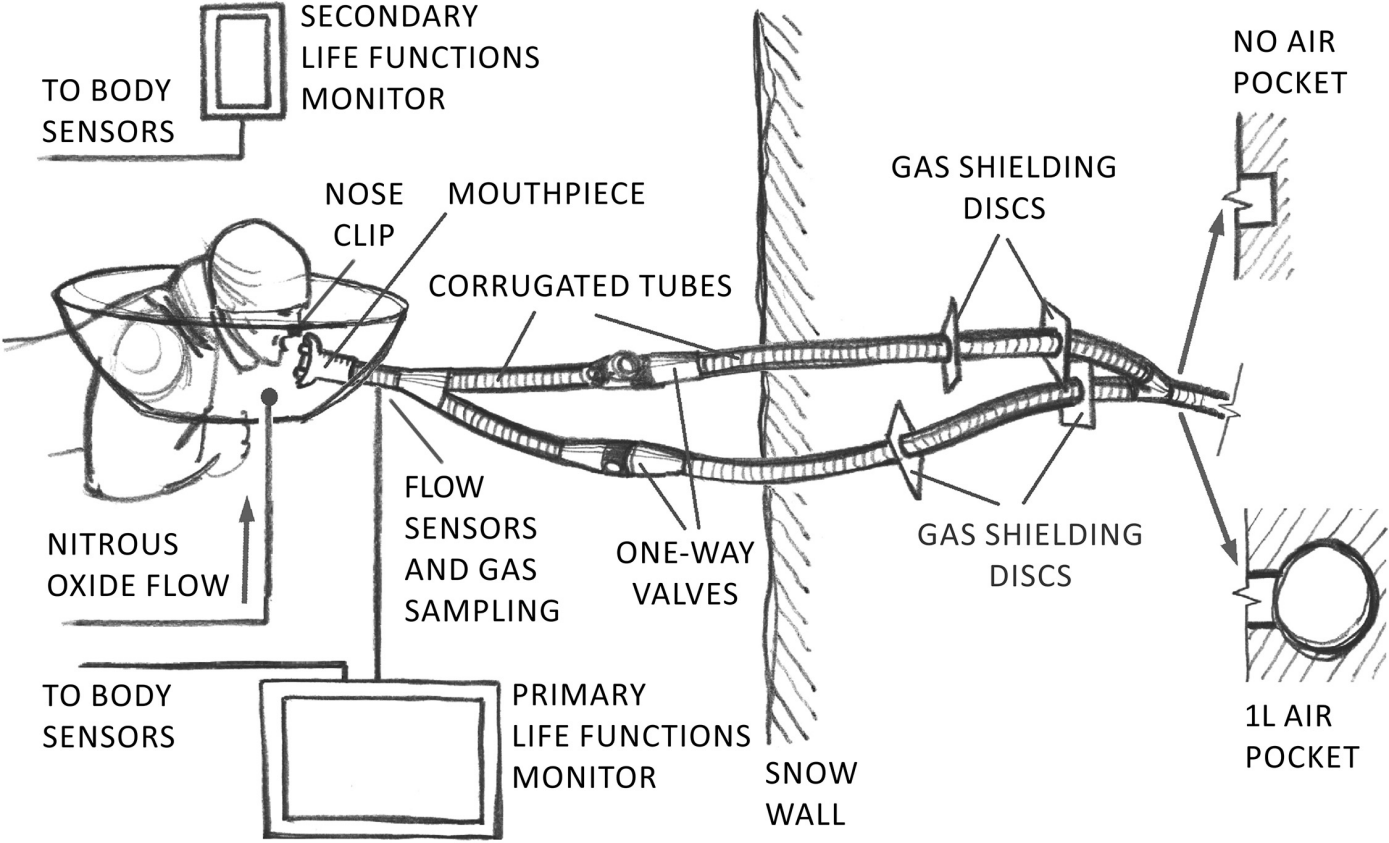
Scheme of the breathing circuit and its installation in the snow. (Illustration: B. Kracmar).

Before starting the breathing experiment, sensors of a life function monitor were attached to the body of the subject. The following parameters were continuously monitored and recorded by Datex-Ohmeda S/5 monitor (Datex-Ohmeda, Madison, WI, USA): Electrocardiography (EKG), heart rate (HR), peripheral blood oxygen saturation (SpO_2_), inspiratory and end-tidal fractions of oxygen (F_I_O_2_, EtO_2_), inspiratory and end-tidal fractions of carbon dioxide (F_I_CO_2_, EtCO_2_), tidal volume (V_T_), breathing frequency (BF), curves of airway pressure (Paw) and airflow (Qaw) measured in the airway opening, concentration of nitrous oxide (N_2_O) in the breathing circuit and non-invasive blood pressure (intermittently in one-minute intervals). The respiratory parameters as of Qaw, BF, Paw and V_T_ were measured using a respiratory sensor D Lite (Datex-Ohmeda, Madison, WI, USA) connected between the mouthpiece and the Y-piece (Fig 2). A spirometry D Lite sensor also provided a gas sampling port for the Datex-Ohmeda S/5 monitor measuring F_I_O_2_, EtO_2_, F_I_CO_2_, EtCO_2_ and N_2_O concentration using an E-CAiOVX (Datex-Ohmeda, Madison, WI, USA) anesthesia and spirometry module.

For safety reasons, another life function monitor Edan M3 (Edan Instruments, Nanshan Shenzhen, China) was used for parallel continuous SpO_2_ and HR measurements in the case that S/5 monitor did not show SpO_2_ or HR values. Both the SpO_2_ sensors (for Datex-Ohmeda S/5 and Edan M3 monitors) were the fingertip sensors attached to the right hand on middle and ring fingers and were well thermally insulated from the ambient environment with a glove. The cuff for the non-invasive blood pressure measurement was attached to the left arm so that it could not affect the SpO_2_ measurement.

The subjects reclined in the prone position on an insulated bed placed in front of the snow wall with already prepared AP or NP described below. Before the breathing experiment, each subject was connected to a mouthpiece allowing measurement of respiratory gasses and ventilatory parameters. After reaching stable ventilatory parameters, especially the steady tidal volume and breathing frequency (after approx. 5 minutes), the subject was connected to the breathing circuit (described below) ending with NP or AP and the breathing experiment was initiated. At the same time, nitrous oxide started to be administered to the vicinity of the head of the subject and the breathing circuit as a tracing gas for possible leak detection. Nitrous oxide is heavier than air and therefore filled the space created around the head of the subject using a plastic foil (see Fig 2). The Datex-Ohmeda S/5 monitor with the anesthesia module was able to detect nitrous oxide present in the breathing circuit when the nose clip of the subject did not sealed perfectly or when the subject inspired other air than that from the breathing circuit (intentionally or by accident).

Another stabilization procedure (approx. 5 minutes) was applied after the end of breathing into snow. This phase lasted until steady readouts, similar to those recorded before the breathing experiment during the stabilization phase, were reached.

Safety of the volunteers during the experiment was assured by advanced monitoring and continuous evaluation of their physiological parameters by a Czech Army military paramedic with a 6-year history of active medical emergency service. Furthermore, a clinician with specialty in anesthesia and emergency medicine continuously assessed the state of the volunteers (based on the continuously measured physiological parameters and assessment of the consciousness of the subjects) and could terminate the experiment at any time. In order to test the consciousness of the volunteers, an investigator continuously asked the volunteer to calculate simple mathematical operations and show the results using fingers.

The breathing experiment would be terminated due to one or more of the following reasons: request of the volunteer; decision made by the anesthesiologist, paramedic or the investigator testing the consciousness of the volunteer; end-tidal partial pressure of carbon dioxide (PaCO_2_) in the expired gas higher than or equal to 60 mm Hg [16]; presence of nitrous oxide in the breathing gas at any measurable concentration; or breathing in the snow over 30 min.

The experiments ended by disconnecting the mouthpiece from the breathing circuit so that the ventilatory parameters could be still measured. Detachment of the mouthpiece and completion of the whole experiment was conducted after the ventilatory parameters stabilized and returned close to the baseline values recorded before the start of the breathing experiment.

### Preparation of the breathing circuit

The subjects were connected to a breathing circuit that ended in the snow by a 1 L cavity for AP or the tube ended directly in the snow during the NP phase as described below. The breathing circuit is depicted in Fig 2. The circuit was designed in order to minimize its dead space; therefore, the circuit comprised separate inspiratory and expiratory branches each equipped with a one-way valve in order to assure the unidirectional gas flow during inspirium and expirium. The one-way valves with a very low flow resistance in the open state (Paedi, Ambu, Ballerup, Denmark) were selected to prevent increase in work of breathing of the subjects. The dead space volume of the bidirectionally ventilated parts of the tubes was 49 mL.

The breathing circuit was placed into the snow wall before the experiment. The snow wall at least 1.5 m high was cut in well-settled snow using a snow-cutter (Proma PSF-6,5/620). A trench 1 m deep, 60 cm wide and 60 cm long was dug for installation of the circuit into the snow. The breathing circuit was then placed into the trench so that the distal end of the circuit, i.e. the distal Y-piece, was at least 40 cm far from the front end of the snow wall. The NP was created by removing the adhesive tape from the end of the Y-piece after it was covered by a 5 cm thick layer of snow to prevent snow from entering the breathing circuit terminal during its burial. The air pocket of 1 L volume was created using a soft mesh of spherical shape supported by a plastic rigid skeleton. The circuit was then buried under snow by snowing the trench up. The snow was tamped to the snow density of 380 ± 14 kg/m^3^.

The gas shielding discs placed tightly on the corrugated tubes prevented gas leak along the tubes.

The life functions monitor Datex-Ohmeda S/5 connected to a laptop computer for data collection was placed in a tent equipped with an electric heater. The temperature in the tent was kept between 5–10°C. The required temperature of the life function monitor and the laptop computer was assured by electric heating foils with input power of 25 W and additional thermal insulation with polyurethane foam. The gas sampling lines of the monitor were supplemented with a heated wire and a polyurethane insulation in order to prevent condensation and freezing of water in the hoses. An external plasma monitor in a transparent plastic box was connected to the life functions monitor and placed outside the tent close to the breathing subject so that the investigators could have immediate information about the subject’s life functions. The secondary life functions monitor Edan M3 was placed outside the tent protected by a transparent plastic box equipped with an electric heating foil. The precision of the gas analysis module of the Datex-Ohmeda S/5 monitor was checked directly on the site before the first breathing experiment every day using a calibrating gas (5% CO_2_, 15% O_2_, balanced by N_2_) from a high-pressure cylinder.

The experiments were recorded on three independent camcorders; the first one continuously recorded the screen of the Datex-Ohmeda S/5 monitor, the second recorded the breathing subject and the third one the overall situation on the site. The video and audio recording was intended as a backup of the data recorded by the laptop computer and the written protocols. This enabled reviewing the situation on the site later during the data processing and evaluation. The investigators communicated via PMR (private mobile radio) strictly adhering to the protocol protecting the double blind design of the study.

In order to quantify the pressure-flow characteristics of the snow against breathing (i.e. the airflow resistance of the snow), we used the standard approach that is used to characterize airflow resistance of anesthesia and respiratory circuits and their parts, bacterial or chemical filters, safety valves etc. This assessment is made by measuring the pressure drop that develops on the measured structure at a steady flow rate of air (60 L/min) [17]. We measured the pressure drop at 60 L/min in each prepared circuit before the breathing experiment started and after the breathing experiment was completed in order to know the initial and final resistance of the snow. The constant flow rate of 60 L/min was generated using an oil-less compressor (Orfi 201/24, Orlík, Prague, CZ), a pressure reducing valve (IR1000, SMC, Tokyo, Japan) and an adjustable pneumatic resistor (ASD230F, SMC, Tokyo, Japan). The system was kept outside before measurement so that the gas used for the measurement had the ambient temperature preventing melting of the snow and changing properties of the air pockets. The overpressure was measured using Testo 512 (Testo, Alton, UK) differential pressure meter.

After the snow resistance measurement, the potential gas leaks along the tubes were checked. Nitrous oxide as a tracing gas was administered into the circuit at the position of Y-piece where the original mouthpiece was exchanged for the source of N_2_O represented by an N_2_O gas cylinder equipped with a pressure reducing valve and a gas flow regulator. The N_2_O flow rate was 20 L/min and N_2_O was administered continuously for 20 s, i.e. the total volume of N_2_O administered into the breathing circuit was 6 L. At the same time, the life functions monitor Datex-Ohmeda S/5 was used to detect N_2_O by sampling gas from a vicinity of the tubes in the snow close to the front end of the snow wall.

Finally, the snow above the breathing circuit was carefully removed so that a possible presence and size of air pocket created during the breathing experiment could be investigated. The snow density of the tamped snow was measured using a ¼ L cutting cylinder with a cutting plate to form an exact cylinder of snow and then determining the weight of the snow cylinder using scales. Then, the density was calculated. The snow sample was cut approx. 10 cm away from the artificial air pocket in the tamped snow so that it would not be affected by melting during the previous breathing experiment.

### Data processing and statistics

From the total sample, only those volunteers who fulfilled the following criteria were included in the final data analysis: they sustained to breathe during both the phases AP and NP for at least 315 seconds and high-quality data were recorded during both the phases. The Kaplan-Meier plot was used to analyze the time to breathing experiment termination for the whole group of subjects and for both the AP and NP phases.

The differences between the recorded physiological parameters (SpO_2_, F_I_O_2_, EtO_2_, F_I_CO_2_, and EtCO_2_) during NP and AP phases were expressed as mean ± standard deviation and presented in the form of a graph. The statistical significance of the difference was tested using two-way ANOVA for repeated measures with Bonferroni post-hoc tests (STATISTICA 7, StatSoft, Tulsa, OK, USA) after the Shapiro-Wilk data normality test. P<0.05 was considered as statistically significant.

In order to compare the differences in breathing efforts between subjects breathing to snow with no air pocket and with the one-liter air pocket due to the flow resistance of the snow, the imposed Pressure-Time Product (iPTP) was calculated. In this case, the term “imposed” refers to the part of the total PTP that is caused by an extra airflow resistance present outside the respiratory system caused by the pressure-flow properties of snow. Inspiratory iPTP_INS_ and Expiratory iPTP_EXP_ were calculated by time integration of the pressure curves measured in the airway opening during inspirium and expirium, respectively, and normalized per minute of breathing, i.e. expressed in Pa s/min. The total iPTP was calculated as the sum of iPTP_INS_ and iPTP_EXP_. The statistical significance of the difference in iPTP was tested using the same tests as the physiological parameters described above.

The initial resistance of the snow against breathing and the final resistance of the snow after the experiment were described using a pressure difference in the breathing circuit against ambient pressure at a steady airflow rate of 60 L/min and expressed as mean ± standard deviation. After the normality of the data was confirmed by Shapiro-Wilk normality test, the paired two-tailed Student’s t-test was used to compare the statistical significance of the initial and final pressure differences between the NP and AP groups.

## Results

**Fig 3:**
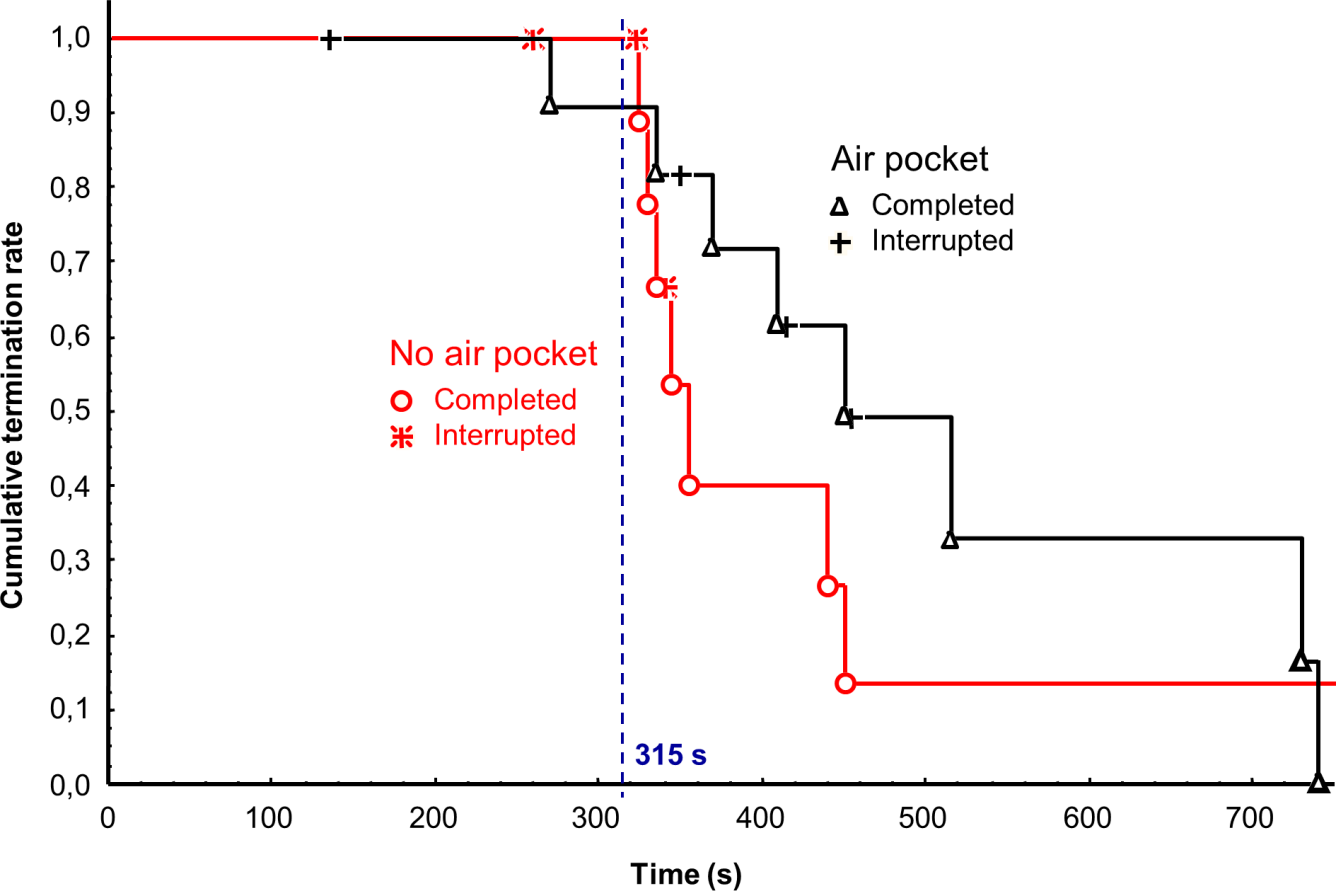
Time to breathing experiment termination for two different conditions: (1) one liter air pocket and (2) no air pocket. The term “completed” means that the subject terminated the experiment upon his own request. The term “interrupted” means that the experiment was interrupted due to other reasons, e.g. the order by the anesthesiologist, critical physiological parameters determined by the life function monitor, or any technical problem.

Ten (83%) of the original number of 12 volunteers completed the whole protocol and were included in data analysis. Two volunteers were excluded due to the following reasons: Breathing of the first volunteer during the AP phase was prematurely terminated after 135 s of the breathing trial according to the order of the anesthesiologist due to the occurrence of frequent bigeminal ventricular extrasystoles. The second volunteer was excluded due to a gas leak detected during the NP breathing phase, and the same volunteer requested to terminate the experiment during the AP phase after 270 s, i.e. earlier than after 315 s requested for the evaluation. There was no harm to the subjects of the experiment. The Kaplan-Meier plots of time to the breathing experiment termination for the whole group of 12 volunteers and for both the AP and NP phases are presented in Fig 3.

The average ambient temperature during the breathing experiments was –4.4 ± 1.3°C (range from –7.6°C to –1.6°C), atmospheric pressure was 91.6 ± 0.4 kPa (range from 90.2 to 92.2 kPa). The well settled snow consisted of grains; the median grain size was 2 mm (1.5 to 2.5 mm). The snow density was 380 ± 14 kg/m^3^. This density corresponds with the moderate dry snow avalanche [18]. The average snow temperature was –5.1 ± 0.7°C (range from –6.1°C to –4.2°C).

**Fig 4:**
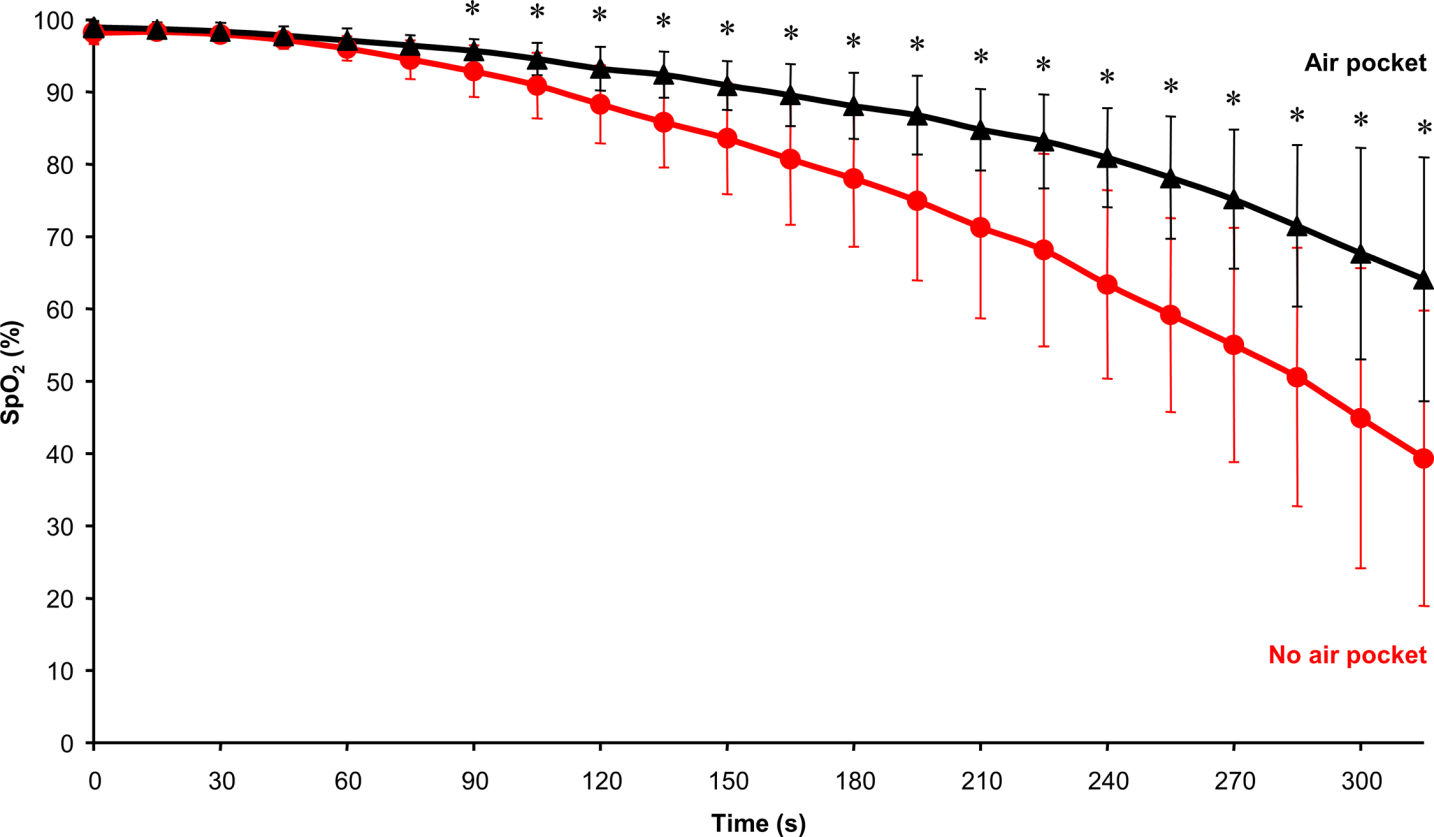
The difference between SpO_2_ during NP and AP phases. The symbol * represents statistically significant differences at p ≤ 0.05.

Existence or the lack of air pocket had a significant effect on the time development of SpO_2_ as demonstrated in Fig 4. After a relatively short initial period when there was no statistical difference between SpO_2_ recorded during AP and NP phases, these two curves started to differ significantly after 90 seconds of the experiment.

**Fig 5:**
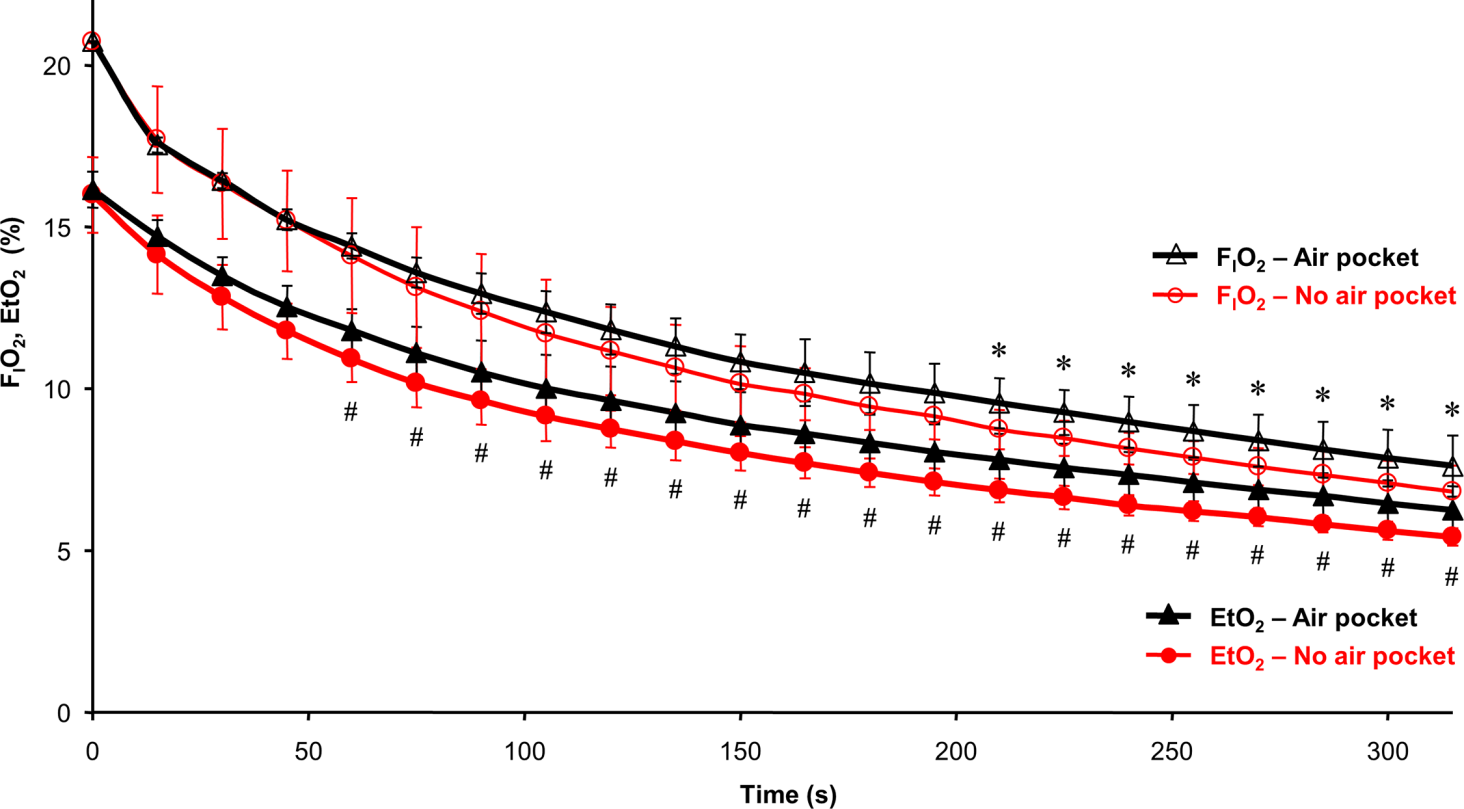
Inspiratory (F_I_O_2_) and end-tidal (EtO_2_) fractions of oxygen in the breathing gas during NP and AP phases. The symbol * represents statistically significant differences in F_I_O_2_ between NP and AP groups; the symbol # represents statistically significant differences in EtO_2_ between NP and AP phases; p ≤ 0.05.

**Fig 6:**
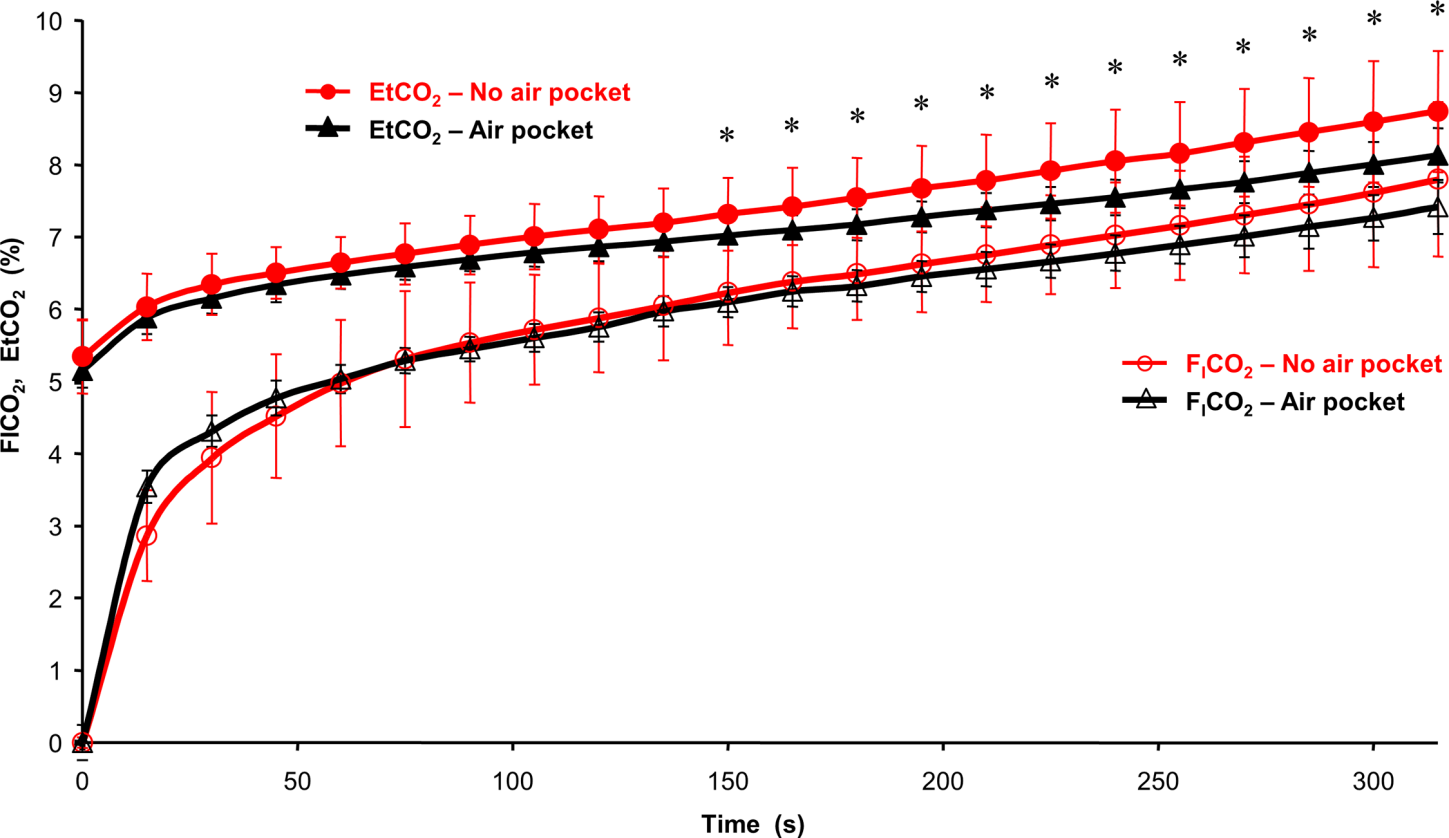
The courses of inspiratory (F_I_CO_2_) and end-tidal (EtCO_2_) fractions of carbon dioxide in the breathing gas during NP and AP phases. The symbol * represents statistically significant differences in EtCO_2_ between the NP and AP groups; p ≤ 0.05.

Inspiratory and end-tidal fractions of oxygen (Fig 5) and carbon dioxide (Fig 6) shared the same trends as that of SpO_2_. However, the difference started to be statistically significant later than for SpO_2_. For both gasses, the significant difference occurred much earlier for end-tidal fractions (60 s for EtO_2_ and 150 s for EtCO_2_) compared to the inspiratory fractions (210 s for F_I_O_2_, whereas F_I_CO_2_ was not significantly different between NP and AP phases).

**Fig 7:**
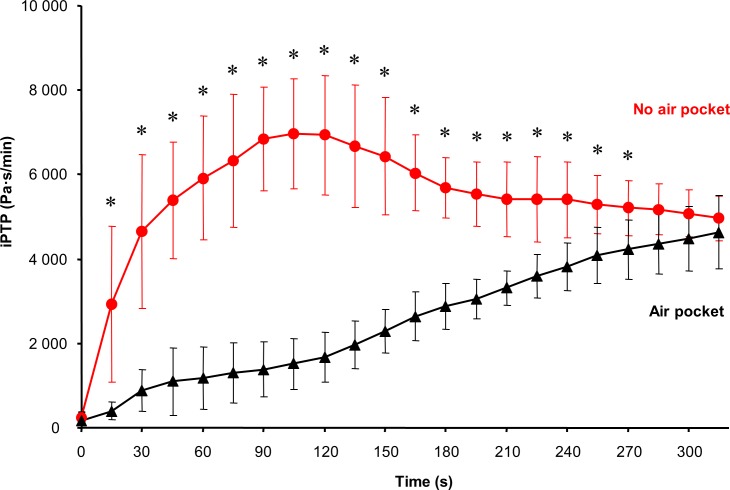
The difference in breathing effort expressed as imposed Pressure-Time Product (iPTP) caused by the flow resistance of snow between no air pocket and one liter air pocket. The symbol * represents the statistical significant differences described by p ≤ 0.05.

The difference in imposed Pressure-Time Product (iPTP) when breathing during AP or NP phases is presented in Fig 7. Immediately after starting the experiment (from the first 15 seconds), there was a significant difference in iPTP between the AP and NP groups. The iPTP was increased several folds during the NP phase. After approximately two minutes, the iPTP during the NP phase started to decrease and to approach the iPTP curve for the AP phase.

**Fig 8:**
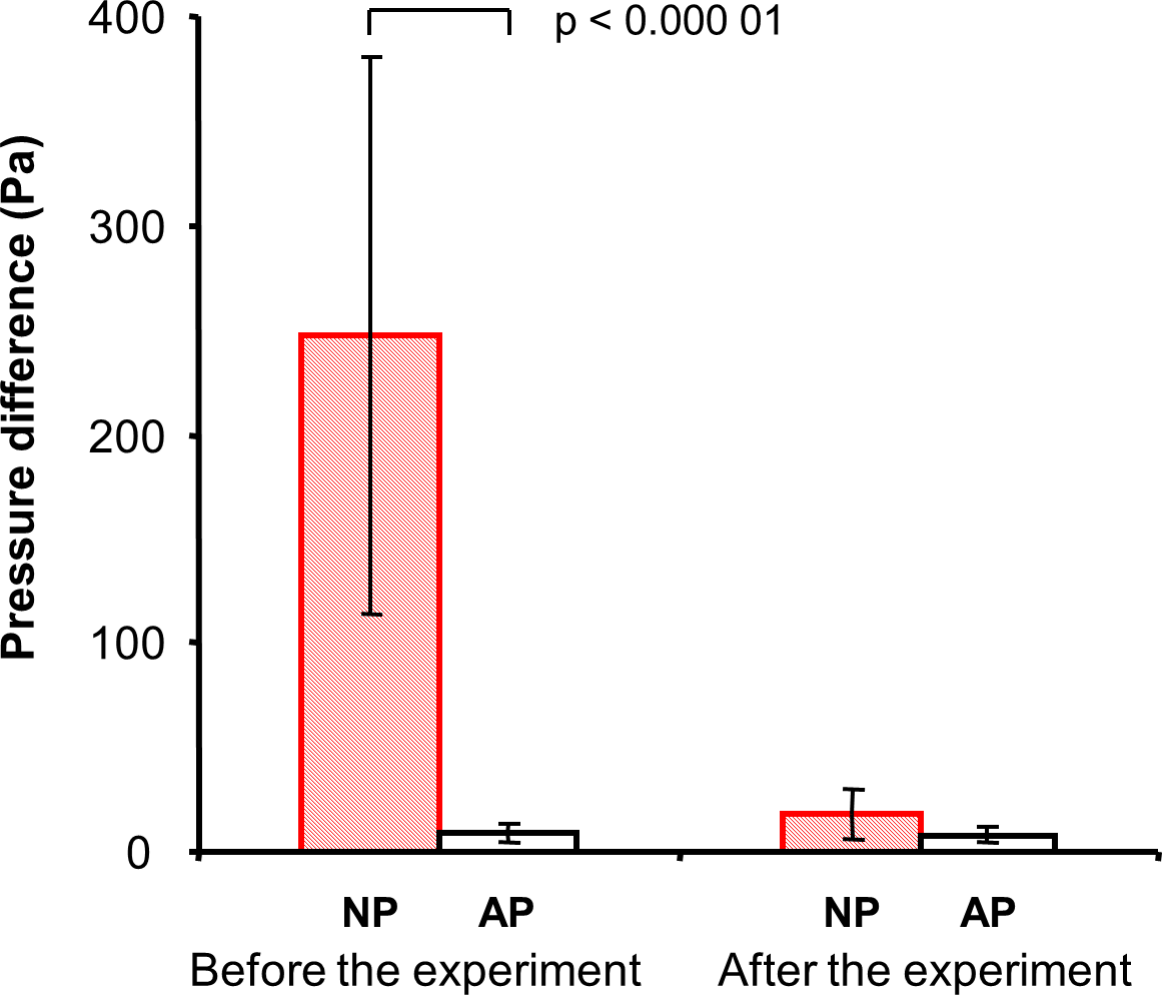
Airflow resistance of snow to breathing expressed as an overpressure in the breathing circuit developed at a constant flow rate of 60 L/min for no air pocket (NP) and one liter air pocket (AP) before and after completing the breathing experiment.

The differences between the groups in the initial pressure-flow characteristics (i.e. resistance) of the snow against breathing are presented in Fig 8. The average overpressure developed in the breathing circuit with no air pocket was 27 fold higher than the average overpressure in the breathing circuit with air pocket at a constant flow rate of 60 L/min. Nevertheless, the final resistances were similar after completing the experiment with no statistically significant difference between them.

After the breathing with no air pocket, a creation of a cavity was documented in all the subjects. The created cavity was of the longitudinal shape with the average diameter of 36 ± 9 mm (range from 24 to 52 mm) and 59 ± 16 mm long (range from 35 to 90 mm). The average volume of the created cavities was 61 ± 34 mL (range from 29 to 127 mL). The snow surrounding the created cavity was wet. No signs of water freezing were observed on the cavity surface in all the subjects.

## Discussion

The main finding of this study was that it is possible to breathe in avalanche snow with no air pocket (0 L volume) in front of patent nose and oral cavity, but breathing under this condition was associated with significantly increased work of breathing. Concerning the differences in respiratory gases between no air pocket and one liter air pocket, the significant difference was first observed for end-tidal values (EtO_2_ and EtCO_2_) and peripheral oxygen saturation (SpO_2_), whereas significant differences in inspiratory fractions occurred much later (for F_I_O_2_) or never (for F_I_CO_2_).

These results suggest two main conclusions: First, the extremely increased work of breathing immediately at the beginning of the experiment in no air pocket seems to be responsible for the rapid changes in end-tidal EtO_2_ and EtCO_2_ values and SpO_2_. This is a consequence of increased work of respiratory muscles and hence increased metabolic rate of the organism, which leads to increased oxygen consumption and carbon dioxide production. The increased consumption of oxygen and production of carbon dioxide further deteriorate the physiological state of the organism.

Second, less noticeable differences in inspiratory oxygen and carbon dioxide fractions in the breathing gas suggest that snow is an environment that is able to supply a certain amount of oxygen and to buffer a certain amount of carbon dioxide.

As previously documented [11], the volume of the air pocket plays an important role in survival of a person buried under avalanche snow. The presence of an air pocket affects the work of breathing. Initially, gas exchange seems to be sufficient in all cases, but over time the significantly increased work of breathing in the absence of an air pocket causes a fast deterioration of the victim.

An interesting finding was that the increase in breathing effort expressed as iPTP was sudden and extensive immediately when breathing into no air pocket was initiated. There was no such change in iPTP when the subjects started to breathe into 1L air pocket (Fig 7). Rather, the increase in iPTP was slow and gradual, corresponding to the gradual increase in tidal volume due to progressive development of hypercapnia related to the rebreathing of expired gas. According to this finding, the real reduction of iPTP can be achieved with air pockets of volumes less than 1 L.

The observed sudden and significant increase in iPTP reflected the high airflow resistance of the snow when no air pocket was present as documented in Fig 8. In this figure, the pressure necessary for assuring the same flow rate (i.e. for overcoming the airflow resistance of snow) differed significantly between NP and AP. This is in concordance with the general principle that a resistance increases with a decrease of the cross-sectional area of the flow and vise versa. The further gradual increase in iPTP during the breathing experiment was caused by hypercapnia-induced increase in tidal volume and flow rate because the pressure drop is proportional to the flow rate according to Hagen-Poiseuille equation.

As the relation between the resistance and the area is not linear according to the same equation, even a small change of the area causes significant changes in airflow resistance when the original area is small. Despite the very significant difference in airflow resistance between NP and AP before the experiment, there was not a statistically significant difference in airflow resistance between NP and AP after the breathing experiment when a relatively small cavity (61 ± 34 mL) reducing the airflow resistance was created.

These results are in concordance with the results of the study published by Brugger et al. [11], who described a non-significant difference between EtCO_2_ values in subjects breathing to 1 L and 2 L air pockets. These volumes (1 or 2 L) are much higher than the air pocket volume necessary for virtually total reduction of breathing effort.

Our study helps to better understand the pathophysiology within the first 5 minutes after avalanche burial in case of no air pocket or presence of minimum (1 L) air pocket in front of patent nose and oral cavity.

A substantial increase in tolerance time under snow achieved using the AvaLung device in the study published by Grissom C.K. et al. [7] can be explained not only by prevention of rebreathing of expired gas by one-way valves and distinct positions of the inspiratory and expiratory outlets, but also by facilitating breathing due to the construction of the device where the inlet port has a large surface area reducing the airflow resistance of snow during breathing.

In this study, we evaluated breathing effort by Pressure-Time Product rather than using Work of Breathing (WoB). PTP is a good indicator of the metabolic work of breathing [19]. Field et al. [20] documented that oxygen consumption of the respiratory muscles is only weakly correlated with the mechanical WOB (the product ΔP·ΔV), whereas it is well reflected by the PTP. PTP takes into account the isometric phase of muscle contraction [21] and represents a good indicator of energy expenditure [22].

The current study has several limitations: First, considerations such as mechanical factors, trauma, psychological stress and, in particular, severe hypothermia, which may be decisive for the outcome in avalanche burial, cannot be experimentally investigated in humans for ethical reasons [11]. Furthermore, the study was designed so that a variety of negative cofactors listed above could be eliminated. As a consequence, the insulated effects of the air pocket presence and imposed breathing effort could be studied. Second, a homogeneous group of volunteers was selected from healthy, well trained and highly motivated young students so that the majority of possible health conditions that might affect the study results could be eliminated. Next, the research was conducted during a stable weather conditions that predetermined a certain physical properties of snow only (e.g. snow type and density, temperature, etc.). Finally, the study was conducted at the low altitude with concomitant higher oxygen partial pressure at the study site. This might influence the presented absolute values of F_I_O_2_, EtO_2_ and SpO_2_, but we believe the trends of these parameters along with the effects studies in this trial were not affected significantly.

## Conclusions

This the first study to document that breathing in snow is possible even without an air pocket in front of patent nose and oral cavity. The limiting factor under these conditions was excessive increase in work of breathing that induces increase in metabolism accompanied by higher oxygen consumption and carbon dioxide production. The presence of an air pocket of 1L volume lowers significantly the work of breathing compared to the absence of an air pocket.

## Supporting Information

S1 File Approval of the study protocol by the Institutional Review Board. (PDF)

S2 File The Consort checklist. (PDF)

## References

[1] Falk M, Brugger H, Adler-Kastner L. Avalanche survival chances. Nature 1994; 368:21 7969398 10.1038/368021a0

[2] Haegeli P, Falk M, Brugger H, Etter HJ, Boyd J. Comparison of avalanche survival patterns in Canada and Switzerland. Canadian Medical Association Journal 2011, 183(7), 789–795. 10.1503/cmaj.101435 21422139 PMC3080528

[3] Boyd J, Haegeli P, Abu-Laban RB, Shuster M, Butt JC. Patterns of death among avalanche fatalities: a 21-year review. Canadian Medical Association Journal 2009; 180:5: 507–512. 10.1503/cmaj.081327 19213801 PMC2645441

[4] McIntosh SE, Grisom CK, Olivares CR, Kim HS, Tremper B. Cause of death in avalanche fatalities. Wilderness & Enviromental Medicine 2007; 18:4: 293–297. 10.1580/07-WEME-OR-092R1.1 18076300

[5] Hohlrieder M, Brugger H, Schubert HM, Pavlic M, Ellerton J, Mair P. Pattern and severity of injury in avalanche victims. High Altitude Medicine & Biology 2007; 8:1: 56–61. 17394418 10.1089/ham.2006.0815

[6] Grissom CK, McAlpine JC, Harmston CH, Radwin MI, Giesbrecht GG, Sholand MB, et al Hypercapnia Effect on Core Cooling and Shivering Treshold During Snow Burial. Aviation, Space, and Environmental Medicine 2008; 79:8: 735–742. 18717110 10.3357/asem.2261.2008

[7] Grissom CK, Radwin MI, Harmston CH, Hirshberg EL, Crowley TJ. Respiration during snow burial using an artificial air pocket. Jama 2000; 283:17: 2261–2271. 10.1001/jama.283.17.2266 10807386

[8] Windsor JS, Hamilton E, Grocott MP, O´Dwyer MJ, Milledge JS. The snow snorkel: A proof of concept study. Wilderness & Enviromental Medicine 2009; 20: 61–65. 10.1580/08-WEME-BR-183.1 19364164

[9] Radwin IM, Grisom CK, Scholand MB, Harmston CH. Normal Oxygenetation and Ventilation during Snow Burial by the Exclusion of Exhaled Carbon Dioxine. Wilderness Enviromental & Medicine 2001; 12: 256–262. 10.1580/1080-6032(2001)012[0256:noavds]2.0.co;2 11769922

[10] Paal P, Strapazzon G, Braun P, Ellmauer PP, Schroeder DC, Suman G, et al Factors affecting survival from avalanche burial–A randomised prospective porcine pilot study. Resuscitation 2013; 84:2: 239–243. 10.1016/j.resuscitation.2012.06.019 22771873

[11] Brugger H, Sumann G, Meister R, Adler-Kastner L, Mair P, Gunga HC. Hypoxia and hypercapnia during respiration into an artificial air pocket in the snow: implications for avalanche survival. Rescuscitation 2003; 58: 81–88. 10.1016/s0300-9572(03)00113-8 12867313

[12] Brugger H, Durrer B, Adler-Kastner L, Falk M, Tschirky F. Field management of avalanche victims. Resuscitation 2001; 51: 7–15. 11719168 10.1016/s0300-9572(01)00383-5

[13] Brugger H, Paal P, Boyd J. Prehospital resuscitation of the buried avalanche victim. High Altitude Medicine & Biology 2011; 12(3): 199–205. 21962062 10.1089/ham.2011.1025

[14] Otis AB. The Work of Breathing. J Physiological Reviews 1954; 34:3: 449–458. 10.1152/physrev.1954.34.3.449 13185751

[15] American Society of Anesthesiologists, INC. New Classification of Physical Status, Anesthesiology, 1963; 24: 111.

[16] Lumb AB. Nunn's Applied Respiratory Physiology. 7th edition Elsevier, 2012.

[17] EN ISO 8835–2. Inhalational anesthesia systems—Part 2: Anaesthetic breathing systems. Brussel: European Comitee for Standardization, 2009.

[18] McClung D, Schaerer PA. The Avalanche Handbook; Mountainers: Seattle, WA, 1993.

[19] Bellani G, Patroniti N, Weismann D, Galbiati L, Curto F, Foti G, et al Measurement of Pressure-Time Product during Spontaneous Assisted Breathing by Rapid Interrupter Technique. Anesthesiology 2007; 106: 484–90. 17325506 10.1097/00000542-200703000-00012

[20] Field S, Sanci S, Grassino A. Respiratory muscle oxygen consumption estimated by the diaphragm pressure-time index. J Appl Physiol 1984; 57: 44–51. 6469790 10.1152/jappl.1984.57.1.44

[21] Sassoon CS, Mahutte CK. Work of breathing during mechanical ventilation, Physiological Basis of Ventilatory Support. Edited by Marini JJ, Slutsky A. New York, Marcel Dekker, 1998 pp. 261–310.

[22] Collett PW, Perry C, Engel LA. Pressure-time product, flow and oxygen cost of resistive breathing in humans. J Appl Physiol 1985; 58: 1263–72. 3988680 10.1152/jappl.1985.58.4.1263

