# Supplementary material for: Work of Breathing into Snow in the Presence versus Absence of an Artificial Air Pocket Affects Hypoxia and Hypercapnia of a Victim Covered with Avalanche Snow: A Randomized Double Blind Crossover Study
Source: PLoS One. 2015 Dec 14;10(12):e0144332. doi: 10.1371/journal.pone.0144332 (PMC4682855; doi:10.1371/journal.pone.0144332)
Supplement: S1 File — (PDF) [file pone.0144332.s001.pdf]

S1 File. Approval of the study by the Institutional review board and the trial study protocol.

UNIVERZITA KARLOVA
FAKULTA TĚLESNÉ VÝCHOVY A SPORTU
José Martího 31, 162 52 Praha 6 – Veleslavín
tel. (02) 2017 1111
http://www.ftvs.cuni.cz/

Žádost o vyjádření etické komise UK FTVS

k projektu výzkumné, doktorské, diplomové (bakalářské) práce, zahrnující lidské účastníky

Název: Změny funkčních parametrů u jedinců v krizové situaci. (Hyperkapnie a difuze plynů pod sněhovou lavinou)
Forma práce: doktorská
Autor/ hlavní řešitel/ PhDr. Mgr. Michal Mašek
Konzultant Ing. Ladislav Sieger, CSc.
Školitel doc. MUDr. Staša Bartůňková, CSc.

Popis projektu
Cílem výzkumu je sledování změn ventilačně-respiračních parametrů v simulované sněhové lavině (vzduchové kapse) a následné použití naměřených dat pro tvorbu matematicko-fyzikálního modelu chování organismu v lavině při narůstající hyperkapnii.
Probandi: studenti z FTVS VO, vojáci z povolání (létající personál a instruktoři přesunů na sněhu a ledu), ostatní dobrovolníci. Místo: Krkonoše, Špindlerův Mlýn, termín: 4.3. - 9.3. 2012.
Zajištění bezpečnosti pro posouzení odborníky: Mezi před-testy bude patřit statická apnoe, spirometrie a dýchání do uzavřeného prostoru, (vytipování zvýšené senzitivity na hypoxii a hyperkapnii). Experiment bude realizován s pomocí pacientského monitoru DATEX Ohmeda, který průběžně monitoruje křivku EKG, srdeční frekvenci, krevní tlak, SpO_2 a veškeré dechové parametry ($EtCO_2$, $FiCO_2$, EtO_2, FiO_2, dechovou frekvenci, dechové objemy, minutovou ventilaci, odpor při výdechu). Dále bude snímána i orientační tělesná teplota probanda, nicméně vzhledem k době trvání se nepředpokládá podchlazení organismu. S probandem bude probíhat neustálá komunikace (musí aktivně reagovat zvednutím ruky, nohy, řešit jednoduché matematické úkoly-ukázat počet prstů, atd.) pro kontrolu mozkové činnosti. Měření bude ukončeno v okamžiku, pokud: a) proband požádá o ukončení, b) nebude reagovat správným způsobem c) některé z měřených parametrů dosáhnou kritických hodnot. K bezpečnostním opatřením patří: přítomnost lékaře a příslušníků Horské služby. K dispozici bude i vozidlo LRD 130 Z (sanita) vybavená pro první pomoc s vyškoleným zdravotníkem.
Projekt byl diskutován se specialisty a zástupci Lékařské komise Českého horolezeckého svazu a Společnosti horské medicíny.
Etické aspekty výzkumu: Účast ve studii je dobrovolná. Výsledky ani osobní data nebudou zneužity.
Informovaný souhlas (přiložen).

V Praze dne..15.2.. 2012 Podpis autora [signature]

Vyjádření etické komise UK FTVS

Složení komise: doc. MUDr. Staša Bartůňková, CSc.
Prof. Ing. Václav Bunc, CSc.
Prof. PhDr. Pavel Slepička, DrSc.
Doc. MUDr. Jan Heller, CSc.

Projekt práce byl schválen Etickou komisí UK FTVS pod jednacím číslem: 077/2012

dne: 17.2.2012

Etická komise UK FTVS zhodnotila předložený projekt a **neshledala žádné rozpory** s platnými zásadami, předpisy a mezinárodní směrnicemi pro provádění biomedicínského výzkumu, zahrnujícího lidské účastníky.

Řešitel projektu splnil podmínky nutné k získání souhlasu etické komise.

razítko školy
UNIVERZITA KARLOVA v Praze
Fakulta tělesné výchovy a sportu
José Martího 31, 162 52, Praha 6
1

podpis předsedy a členů EK

Sylabus výzkumného projektu

A: Základní údaje o výzkumném projektu:

Název projektu: **Hyperkapnie a difuze plynů pod sněhovou lavinou**

Název anglicky: Hypercapnia and Gas Exchange Under the Simulated Avalanche Snow

Typ studie:	X intervenční<br>☐ observační	X prospektivní<br>☐ retrospektivní
Půjde o práci:	X s osobami	☐ s laboratorními zvířaty

(V závislosti na typu testovaných subjektů vyplňte buď část B, nebo část C tohoto sylabu)

Cíl projektu, testovaná hypotéza:

Cílem výzkumu je sledování změn ventilačně-respiračních parametrů zdravých dobrovolníků v simulované sněhové lavině (vzduchové kapse) a následné použití naměřených dat pro tvorbu matematicko-fyzikálního modelu chování organismu v lavině při narůstající hyperkapnii.

Přínos projektu v technické, diagnostické, léčebné oblasti, pro lékařské poznání nebo individuální subjekt hodnocení:

Cílem celého projektu, jehož součástí je předkládaný výzkum na dobrovolnících, je poznat základní rizika spojená s výměnou plynů při dýchání v lavinovém sněhu za komplexního monitorování ventilačních a fyziologických parametrů a nalezení závislosti těchto parametrů na fyzikálních vlastnostech sněhu, velikosti sněhové kavity a dalších parametrech. Jedná se o jediný experiment prováděný v podmínkách detailního a komplexního monitorování a vztažený k fyzikálním vlastnostem sněhu. Výzkumníci předpokládají, že velikost vzduchové kapsy významně souvisí s dechovou prací a s odporem proudícího vzduchu při dýchání. Cílem studie je zjistit efekt velikosti vzduchové kapsy na výměnu plynů a dechovou práci u probandů dýchajících do simulované sněhové laviny a otestovat, zda je možné dýchat do sněhu s nulovým objemem vzduchové kapsy.

B: Charakteristika souboru subjektů (osob) klinického hodnocení:

Počet: 12	Věková struktura: 20 – 30 let
Pohlaví: M	Charakteristika skupiny: dobrovolníci budou studenti FTVS UK, Vojenský obor.
Odměna za účast: 0	Jiné: ---
Doba trvání celého projektu (datum od-do):<br>4. – 9. 3. 2012	Doba trvání pro jeden subjekt hodnocení:<br>30 min experiment + příprava, předtesty a poučení

Popis nakládání se subjekty, popis intervence, odebírání vzorků apod.:

V přednášce (bezpečnostním poučení) budou probandi seznámeni s problematikou přežití pod lavinou a s významem prováděného měření. Bude vysvětlena situace při hypoxemii a hyperkapnii a také podrobný průběh testování.

Po absolvování předtestů bude každý proband dýchat do vzduchové kapsy o definovaném

objemu, který nebude proband ani zkoušející znát. V rámci experimentu nebude proband zasypán sněhem. Bude ležet na sněhu na nosítkách s karimatkou, obličejem dolů. V ústech bude mít náustek, který umožní dýchání do prostoru vzduchové kapsy. Zde bude docházet ke konvekci vzduchu ze sněhu do kapsy a difuzi CO_2 z kapsy do sněhu. Měření bude realizováno pomocí jednocestných ventilů tak, aby došlo k nádechu ze sněhové kapsy a výdechu opět zpátky do sněhové kapsy. Exspirované CO_2 začne postupně způsobovat nárůst CO_2 i v inspirovaném vzduchu a snižování obsahu O_2 v inspirované směsi. S rostoucím časem bude dýchání stále obtížnější. Předpokládaná doba měření bude 3 - 15 minut (závislost na objemu a ploše sněhové kapsy a poréznosti sněhu), maximálně však 30 minut.

Liší se projekt od standardního postupu v denní praxi: X ANO ☐ NE
V případě, že ano, popište rozdíly:
Jedná se o uměle připravenou experimentální situaci detailně popsanou výše.

Možná rizika, nesnáze a obtíže pro subjekt hodnocení (včetně etických):

Pro subjekt hrozí rizika způsobená hladinou krevních plynů mimo běžný fyziologický rozsah. Pro minimalizaci těchto rizik a zamezení dosažení nebezpečných hodnot krevních plynů jsou zavedena opatření popsaná v následujícím odstavci.

Experimentu se mohou zúčastnit pouze jedinci se zdravotní klasifikací ASA I bez historie kouření.

Mezi vyřazující kritéria patří zejména jakákoliv respirační a kardiovaskulární onemocnění, a to jak chronická, tak akutní, Tiffenau index menší než 0,70.

Jak je zajištěna bezpečnost testovaného subjektu:

Mezi diagnostické předtesty (vstupní vyšetření) bude patřit spirometrie, EKG, krevní tlak, anamnéza a ohodnocení zdravotního stavu anesteziologem.

Experiment bude realizován s pomocí pacientského monitoru DATEX Ohmeda, který průběžně monitoruje křivku EKG, srdeční frekvenci, krevní tlak, SpO_2 a veškeré dechové parametry ($EtCO_2$, $FiCO_2$, EtO_2, FiO_2, dechovou frekvenci, dechové objemy, minutovou ventilaci, odpor při výdechu). Bude použit ještě jeden nezávislý přístroj na monitorování saturace krve kyslíkem SpO_2. Bude snímána i orientační tělesná teplota probanda, nicméně vzhledem k době trvání se nepředpokládá podchlazení organismu.

S probandem bude probíhat neustálá komunikace (musí aktivně reagovat zvednutím ruky, nohy, řešit jednoduché matematické úkoly-ukázat počet prstů, atd.) pro kontrolu mozkové činnosti. Měření bude ukončeno v okamžiku, pokud: a) proband vzdá experiment, požádá o ukončení či jinak od studie odstoupí, b) nebude reagovat správným způsobem na testy c) některé z měřených parametrů dosáhnou kritických hodnot, d) na příkaz anesteziologa či zkoušejícího sledující stav probanda.

Jako kritická hodnota byla stanovena následující veličina:
Parciální tlak CO_2 ve vydechovaném plynu ($EtCO_2$) dosáhne 60 mm Hg.

K bezpečnostním opatřením patří: trvalá přítomnost lékaře (zajištěná memorandem o spolupráci se Společností horské medicíny (SHM), která se zavázala mj. k zajištění

odborného zdravotního personálu a dohledu při realizaci měření v terénu. Dále budou přítomni příslušníci Horské služby.<br><br>K dispozici bude i vozidlo LRD 130 Z (sanita) vybavená pro první pomoc s vyškoleným zdravotníkem.<br><br>Projekt byl diskutován se specialisty a zástupci Lékařské komise Českého horolezeckého svazu a Společnosti horské medicíny.	
Kdo ponese náklady na odškodnění v případě poškození subjektu hodnocení:<br><br>FTVS UK	
C: Charakteristika souboru laboratorních zvířat: ----	
Druh laboratorních zvířat:	Počet:
Doba trvání celého projektu (datum od-do):	Doba trvání pro jeden subjekt hodnocení:
Jak bude zajištěna péče o zvířata v průběhu experimentu:	
D: Doplňující informace k výzkumnému projektu:	

Trial Study Protocol

A: Basic information about the research project:	
Title in English: **Hypercapnia and Gas Exchange Under the Simulated Avalanche Snow**<br><br>Title in Czech: **Hyperkapnie a difuze plynů pod sněhovou lavinou**	
Type of the study: X interventional ☐ observational	X prospective ☐ retrospective
Subjects: X human subjects	☐ animals
The aim of the study, tested hypothesis:<br><br>The aim of the study is to investigate respiratory parameters of a person in the simulated avalanche snow and consequent use of the measured data for development of a mathematical-physical model of breathing during increasing hypercapnia in the avalanche.	
Contributions of the project to the diagnosis, technical and medical sciences and to the subjects involved:<br><br>The study is aimed at studying physiological conditions and development of breathing parameters of a person breathing in the simulated avalanche snow. Presence of an air pocket and its size play an important role in survival of victims buried in the avalanche snow. Even small air pockets facilitate breathing, yet they do not provide a significant amount of fresh air for breathing. The investigators hypothesize that the size of the air pocket significantly affects the airflow resistance and work of breathing. The aim of the study is to investigate the effect of the air pocket volume on gas exchange and work of breathing in subjects breathing into the simulated avalanche snow and to test, whether it is possible to breathe with zero air pocket.	

B: Characteristics of the subjects in the project:	
Number: 12	Age: 20 – 30 years
Gender: Males	Characteristics of the group: volunteers from the Czech Army forces, studying at the Military Department of the Faculty of Physical Education and Sport, Charles University in Prague.
Payment: 0	Others: ---
Duration of the whole projects (date from-till):<br>March 4. – 9., 2012	Duration for each single subject:<br>30 minutes of experiment + preparation, examination and safety instructions
Description of procedures with the subjects, interventions, collection of samples, etc.:<br><br>In the introductory lecture (safety instructions), the subjects will be informed about current knowledge about survival in the avalanche snow and with the reasons of the research.	

Situation during hypercapnia and hypoxia will be explained, detailed protocol will be discussed.

After the entrance examination, each volunteer will be breathing into the snow air pocket with a defined volume unknown to the subject and the investigator. During the experiment, the volunteer will not be covered with the snow. The volunteer will lay in prone position on an insulated bed. He will have a mouthpiece in his mouth allowing breathing into the snow where the gas exchange will take place: diffusion of CO_2 from the air pocket to the snow and O_2 from the snow to the air pocket. A system of one-way valves will be used so that the inspirium would be conducted directly from the air pocket and expirium directly into the air pocket. Expired CO_2 will start to accumulate and increase CO_2 fraction in the inspired gas. The oxygen fraction will decrease likewise. Breathing will become more and more difficult. The expected duration of the experiment is 3 - 15 minutes (in dependency of the air pocket size and area and the show properties); the maximum duration is 30 minutes.

Does the procedure differ from the usual procedures?: X Yes ☐ No

If yeas, please, describe:

The project is an experimental research study of breathing in the simulated avalanche snow. The details are described above.

Potential risks, problems, etc., for the subjects (including ethical):

There are risks associated with the levels of the blood gases outside the normal physiological range. To prevent any harm and to assure safety of the subjects, the measures described in the next section will be adopted.

All subjects will be healthy and fit, classified as ASA I, all without a smoking history.

The exclusion criteria will be Tiffeneau Index less than 0.70 and any cardiovascular or respiratory condition.

How the safety of the volunteers will be assured:

The entrance examination, completed before the start of the study, will include these tests: electrocardiography, blood pressure, spirometry (MSP1, Mesit, Uherske Hradiste, CZ), and assessment of the health conditions and family anamnesis by a physician with a specialty in anesthesia and critical care.

The following parameters will be continuously monitored and recorded by Datex-Ohmeda S/5 monitor (Datex-Ohmeda, Madison, WI, USA): Electrocardiography (EKG), heart rate (HR), peripheral blood oxygen saturation (SpO_2), inspiratory and end-tidal fractions of oxygen (F_IO_2, EtO_2), inspiratory and end-tidal fractions of carbon dioxide (F_ICO_2, $EtCO_2$), tidal volume (V_T), breathing frequency (BF), curves of airway pressure (Paw) and airflow (Q) measured in the airway opening, concentration of nitrous oxide (N_2O) in the breathing circuit and non-invasive blood pressure (intermittently in one-minute intervals). For safety reasons, another life function monitor Edan M3 (Edan Instruments, Nanshan Shenzhen, China) will be used for parallel continuous SpO_2 and HR measurements in the case that S/5 monitor will not show SpO_2 or HR values.

In order to test the consciousness of the volunteers, an investigator will continuously ask the volunteer to calculate simple mathematical operations and show the results using fingers.<br><br>The breathing experiment will be terminated due to one or more of the following reasons: request of the volunteer; decision made by the anesthesiologist, paramedic or the investigator testing the consciousness of the volunteer; end-tidal partial pressure of carbon dioxide ($PaCO_2$) in the expired gas higher than or equal to 60 mm Hg; or breathing in the snow over 30 minutes.<br><br>As a critical value for termination of the experiment in a subject, the partial pressure of CO_2 in the expired gas ($EtCO_2$) higher than 60 mm Hg was selected.<br><br>The safety measures will include also: continuous evaluation of physiological parameters and a status of the volunteers by an experienced clinician with specialty in anesthesia and emergency; unlimited availability of an emergency ambulance car LRD 130 Z fully equipped for the first aid and with a trained paramedic.<br><br>The project was consulted with experts from the Medical section of the Czech mountaineering association, and from the Czech society for mountain medicine.
Who will reimburse the costs associated with a possible damage to the subjects:<br><br>Charles University in Prague, FTVS.

C: Charakteristics of the group of animals: ----	
Druh laboratorních zvířat:	Počet:
Doba trvání celého projektu (datum od-do):	Doba trvání pro jeden subjekt hodnocení:
Jak bude zajištěna péče o zvířata v průběhu experimentu:	

D: Additional information about the project:
